# A new species of the genus *Hyalella* (Crustacea, Amphipoda) from northern Mexico

**DOI:** 10.3897/zookeys.942.50399

**Published:** 2020-06-18

**Authors:** Aurora Marrón-Becerra, Margarita Hermoso-Salazar, Gerardo Rivas

**Affiliations:** 1 Posgrado en Ciencias del Mar y Limnología, Universidad Nacional Autónoma de México; Av. Ciudad Universitaria 3000, C.P. 04510, Coyoacán, Ciudad de México, México Universidad Nacional Autónoma de México México Mexico; 2 Facultad de Ciencias, Universidad Nacional Autónoma de México; Av. Ciudad Universitaria 3000, C.P. 04510, Coyoacán, Ciudad de México, México Universidad Nacional Autónoma de México México Mexico

**Keywords:** Durango, freshwater amphipod, Nearctic region, scud, taxonomy

## Abstract

A new species, *Hyalella tepehuana***sp. nov.**, is described from Durango state, Mexico, a region where studies on *Hyalella* have been few. This species differs from most species of the North and South American genus *Hyalella* in the number of setae on the inner plate of maxilla 1 and maxilla 2, characters it shares with *Hyalella faxoni* Stebbing, 1903. Nevertheless, *H. faxoni*, from the Volcan Barva in Costa Rica, lacks a dorsal process on pereionites 1 and 2. Also, this new species differs from other described *Hyalella* species in Mexico by the shape of the palp on maxilla 1, the number of setae on the uropods, and the shape of the telson.

## Introduction

Covich et al. (2009) recorded seven species of the amphipod *Hyalella* Smith, 1874 for North America (including northern Mexico) and emphasized that there were probably one or more undescribed species. Until now, there are 12 formally described species in North America, nine of them from the United States of America: *Hyalella texana* Stevenson & Peden, 1973; *H. montezuma* Cole & Watkins, 1977; *H. longicornis* Bousfield, 1996; *H. muerta* Baldinger, Shepard & Threloff, 2000; *H. sandra* Baldinger, Shepard & Threloff, 2000; *H. meraspinosa* Baldinger, 2004; *H. spinicauda* Soucek & Lazo-Wasem, 2015; *H. wellborni* Soucek & Lazo-Wasem, 2015; *H. wakulla* Drumm & Knight-Gray, 2019; and *Hyalella cheyennis* Bueno, Oliveira & Wellborn, 2019.

Mexico is in the transition zone between two biogeographic regions: the Nearctic and the Neotropical regions. In the Neotropical region, the genus has been found to be highly diverse, with three species in Mexico, three species in the Caribbean region, two species in Central America, and more than 60 species in South America (Marrón-Becerra et al. 2018; Horton et al. 2019). Of the three species in Mexico, the type locality of *Hyalella azteca* (De Saussure, 1858) is in Veracruz, and the type localities of *Hyalella cenotensis* Marrón-Becerra, Hermoso-Salazar & Solis-Weiss, 2014 and *Hyalella maya* Marrón-Becerra, Hermoso-Salazar & Solis-Weiss, 2018 are in the Yucatan Peninsula. In northern Mexico, the taxonomic status of *Hyalella* populations is unknown, and the few records of *H. azteca*, identified by Rodríguez-Almaraz et al. (2014), from from Parque Nacional Cumbres de Monterrey in Nuevo Leon state requires morphological confirmation. Most of the studies on amphipods in northern Mexico are focused on the stygobitic environment (e.g. Holsinger 1973). Herein, we record and formally describe for the first time an epigean amphipod, *Hyalella tepehuana* sp. nov., from Durango state. This new species is the first epicontinental freshwater amphipod described in the Nearctic Region of Mexico.

## Materials and methods

**Figure 1.**
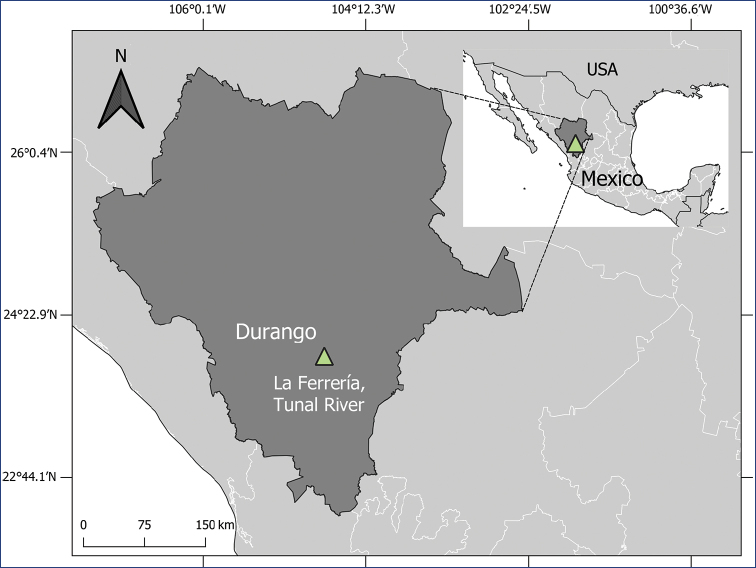
Type locality. Tunal River, La Ferrería, Durango state, Mexico (23°57.905'N, 104°39.817'W).

The material was collected using a net with fine, 250 µm mesh on aquatic vegetation in the Tunal River in Durango state, Mexico (Fig. 1). This river belongs to San Pedro hydrological basin and flows to the Pacific Ocean.

The body parts of the collected material were dissected and mounted: semi-permanent slides were mounted on glycerol, and permanent slides on Entellan, a synthetic resin. The terminology used for the setae follows that of Zimmer et al. (2009). The morphological description includes intraspecific variation.

The type material was deposited in the Colección Nacional de Crustáceos (CNCR), Instituto de Biología, Universidad Nacional Autónoma de México (UNAM).

Scanning electron micrographs were taken from paratypes (one female and one male) with a Hitachi SU1510 scanning electron microscope at the Laboratory of Microscopy and Photography of Biodiversity I, Instituto de Biología, UNAM.

We compared our specimens with the lectotype and syntype material (now paralectotype) of *Amphitoe aztecus*, originally collected by De Saussure (1858) and in the Muséum d’Histoire Naturelle, Geneva, Switzerland. This material had been redescribed as *H. azteca* lectotype (no catalogue number assigned) by González and Watling (2002a).

We present a key of the species from North America, Central America, and Caribbean region. However, *Hyalella sapropelica* Brehm, 1939 is excluded because the short description and incomplete drawings, make it is necessary to redescribe this species. In the key, we retain the subgenus Hyalella (Hyalella), proposed by Bousfield (1996); Baldinger (2004) considered Bousfield’s classification to be an artificial grouping of species with no curved spine in the male uropod 1, the propodus in gnathopod 1 mainly hammer-shaped, five pairs sternal gills, the ramus of uropod 3 “elongated”, and the telson mainly with paired setae. Nevertheless, we emphasize this classification is no longer accepted, and a revision of the characters and relationships proposed by Bousfield (1996) need revision.

## Taxonomy

### Order Amphipoda Latreille, 1816

Family Hyalellidae Bulycheva, 1957

Genus *Hyalella* Smith, 1874

#### Hyalella tepehuana sp. nov.

Taxon classificationAnimaliaAmphipodaHyalellidae

C2DD8C78-67C1-518D-B3E1-50185B66BAF3

http://zoobank.org/0DF61E79-825B-4882-B1F2-A0DE3FA90503

**Figure 2.**
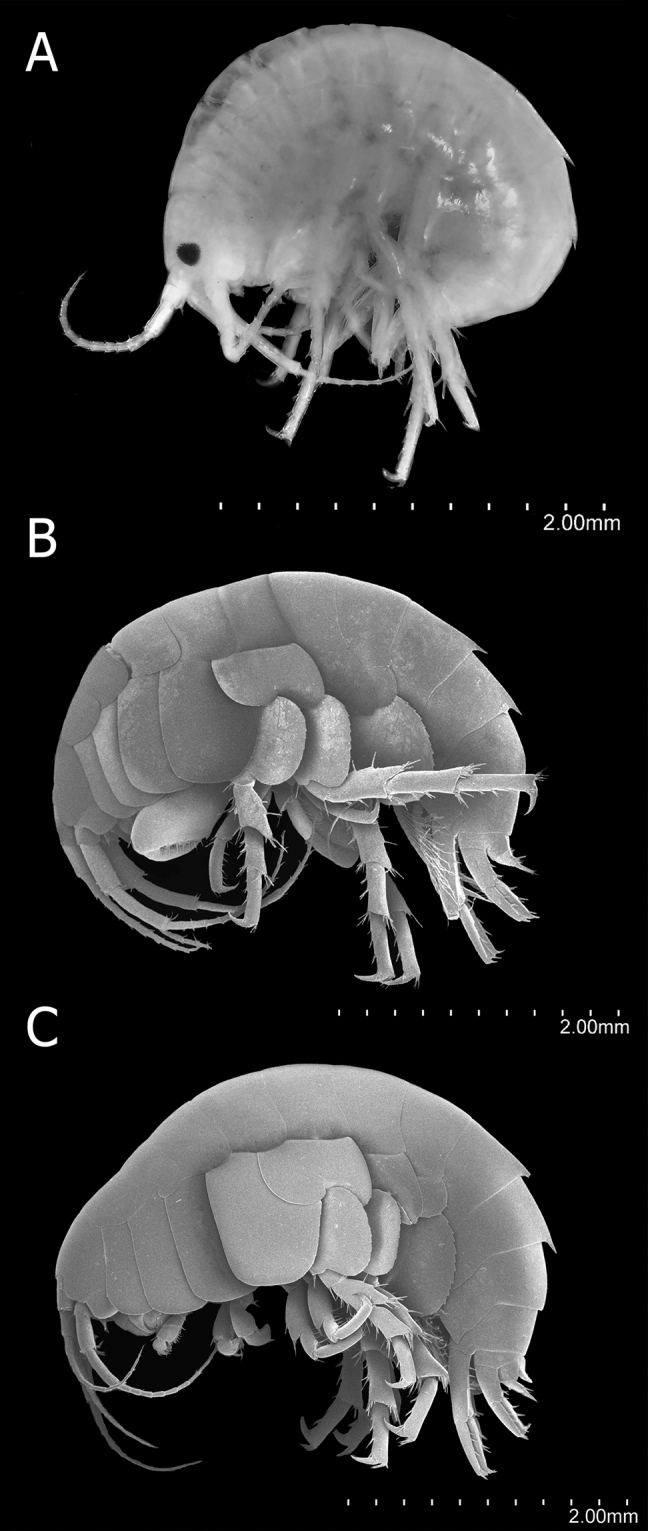
*Hyalella tepehuana* sp. nov. lateral habitus **A** male holotype 5.4mm **B** male paratype 5.6 mm **C** female paratype 5.6 mm. Scale bars: 2 mm.

**Figure 3.**
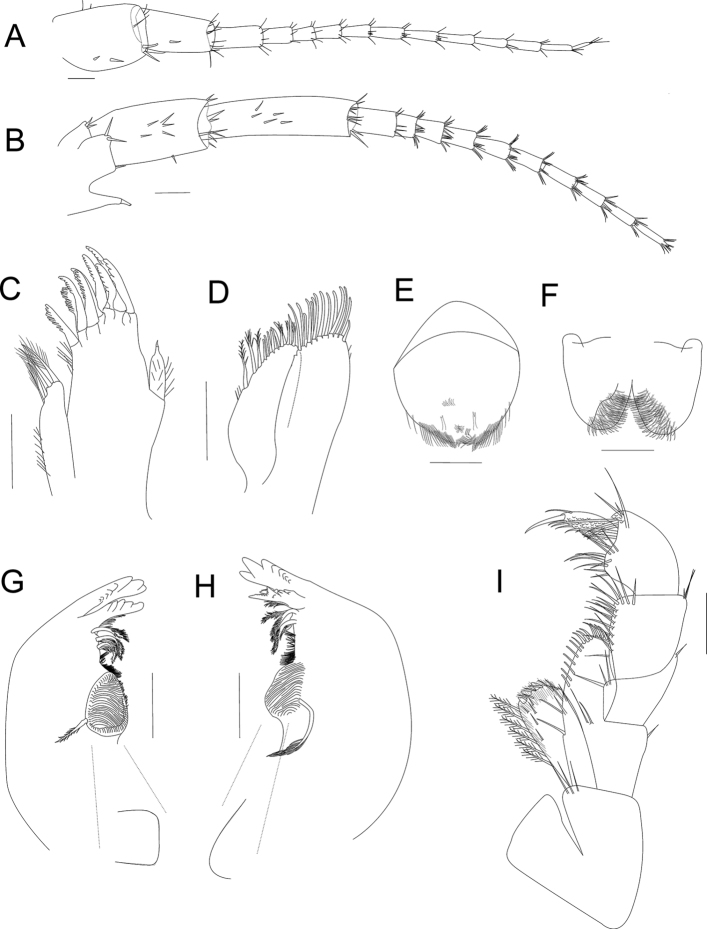
*Hyalella tepehuana* sp. nov., male antennae **A** antenna 1 **B** antenna 2. Buccal parts **C** maxilla 1 **D** maxilla 2 **E** upper lip **F** lower lip **G** left mandible (dotted line shows the form and angle of molar in lateral view) **H** right mandible (dotted line shows the form and angle of molar in lateral view) **I** maxilliped. Scale bars: 100 µm.

**Figure 4.**
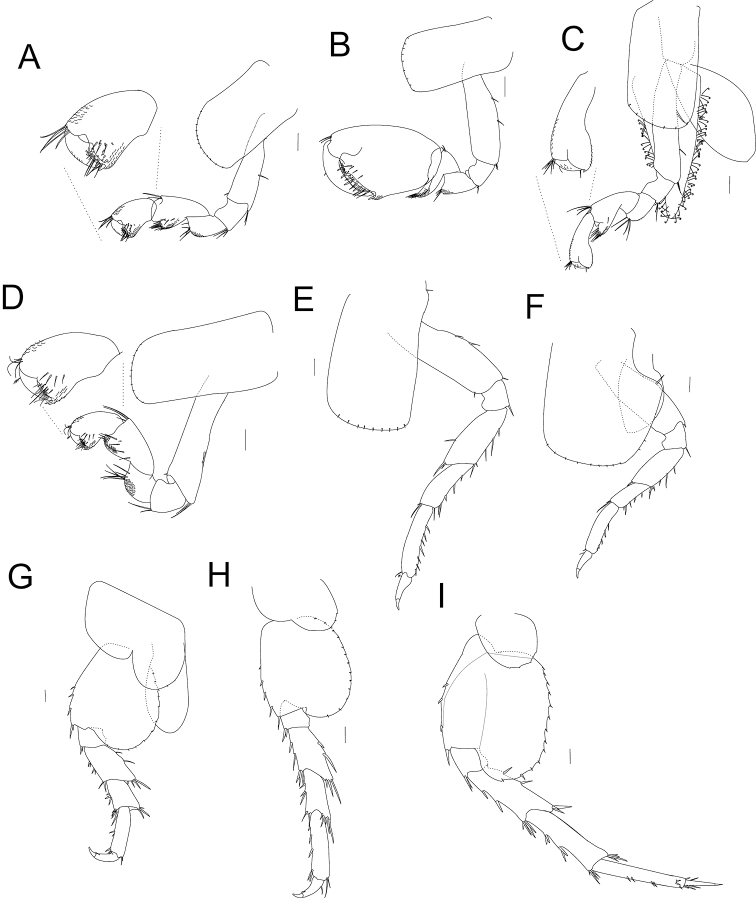
*Hyalella tepehuana* sp. nov. gnathopods **A** male gnathopod 1 **B** male gnathopod 2 **C** female gnathopod 2 **D** female gnathopod 1. Male pereiopods **E** pereiopod 3 **F** pereiopod 4 **G** pereiopod 5 **H** pereiopod 6 **I** pereiopod 7. Scale bars: 100 µm.

**Figure 5.**
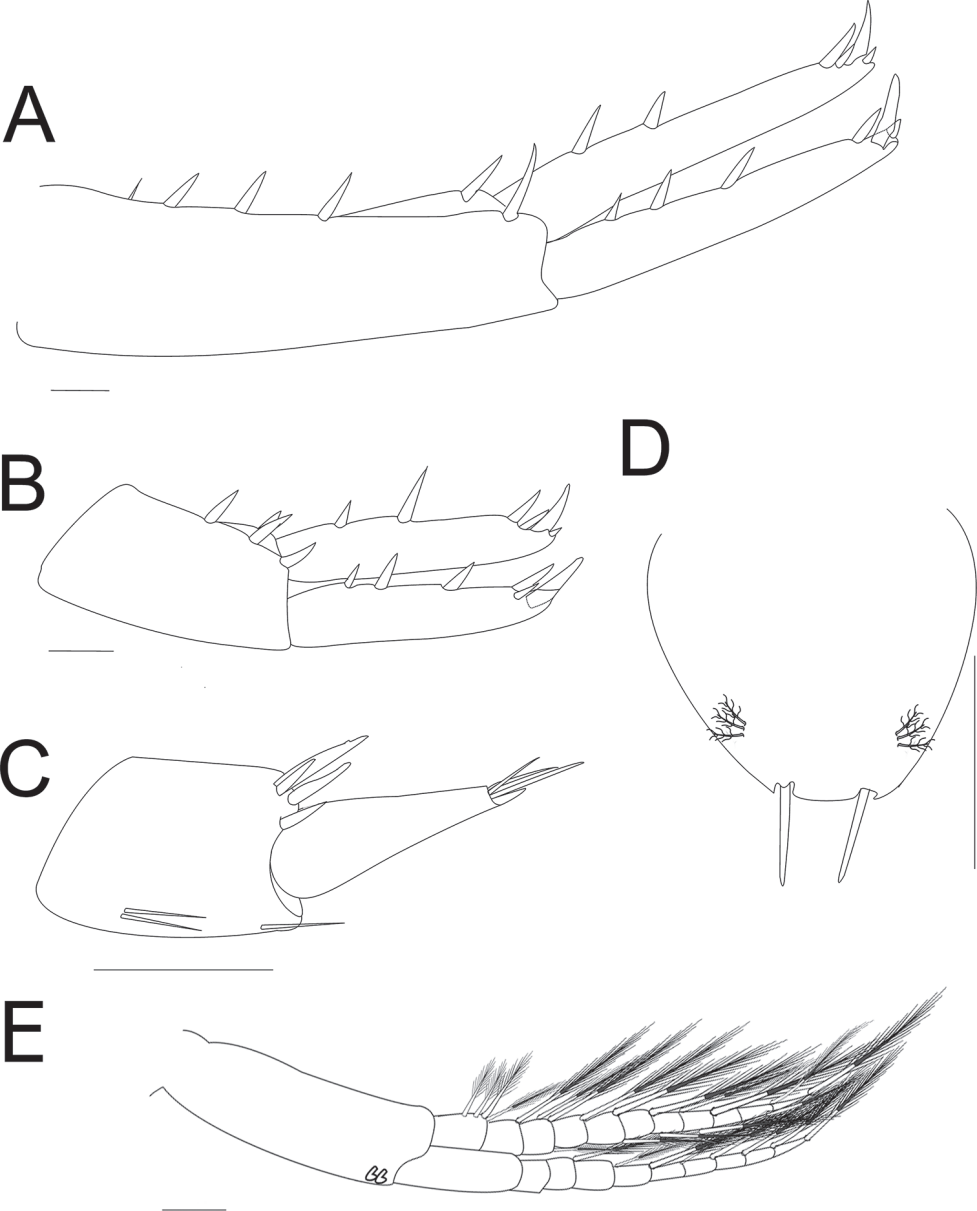
*Hyalella tepehuana* sp. nov. Male uropods **A** uropod 1 **B** uropod 2 **C** uropod 3 **D** telson **E** Pleopod 1. Scale bars: 100 µm.

**Figure 6.**
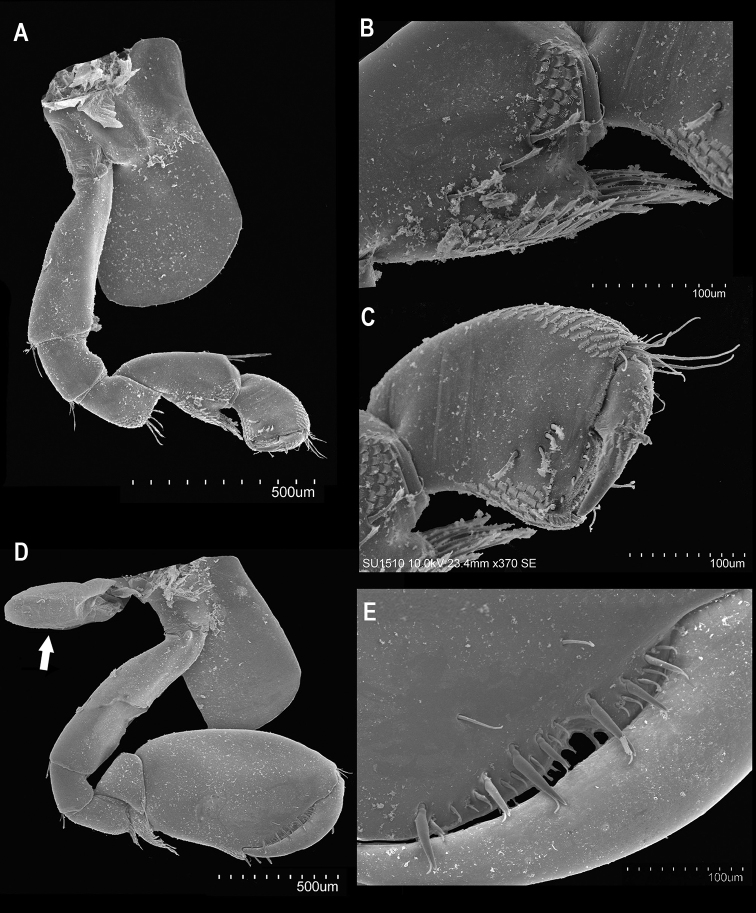
*Hyalella tepehuana* sp. nov. Male gnathopods **A** gnathopod 1 inner face **B** carpus gnathopod 1 inner face **C** propodus and dactyl gnathopod 1 inner face **D** gnathopod 2 inner face (arrow shows coxal gill) **E** palm gnathopod 2 inner face.

**Figure 7.**
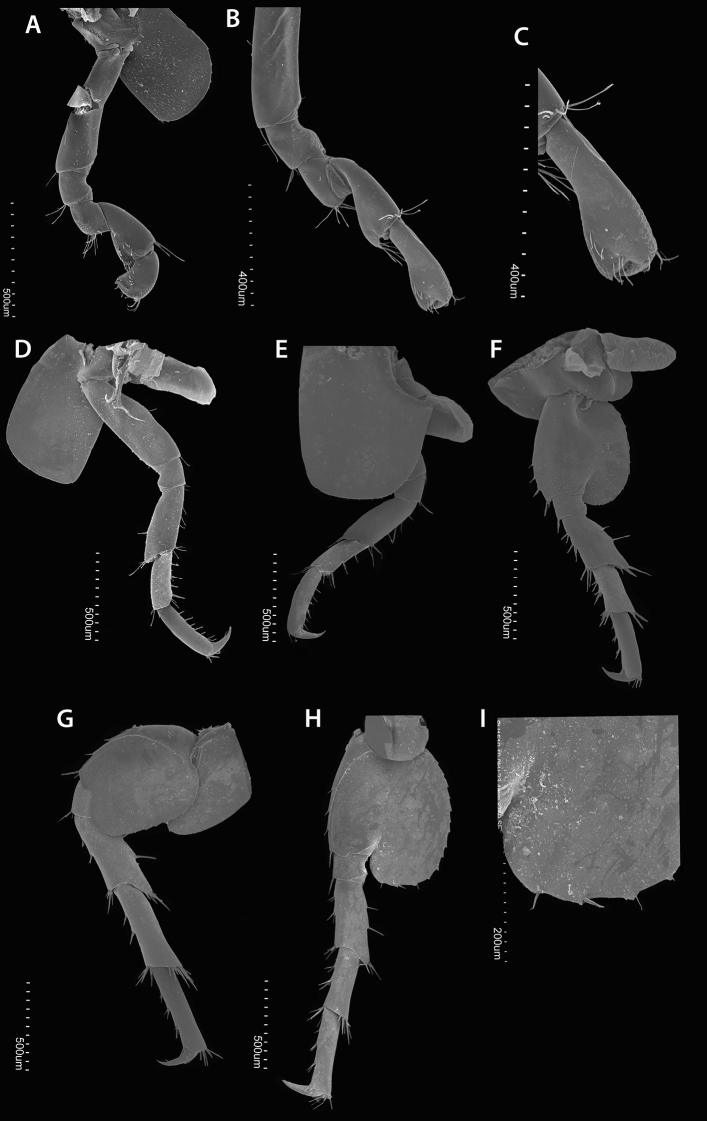
*Hyalella tepehuana* sp. nov. Female gnathopods **A** gnathopod 1 **B** gnathopod 2 **C** propodus gnathopod 2. Male pereiopods **D** pereiopod 3 **E** pereiopod 4 **F** pereiopod 5 **G** pereiopod 6 **H** pereiopod 7 **I** pereiopod 7 basis posterior lobe, distal margin stout setae.

**Figure 8.**
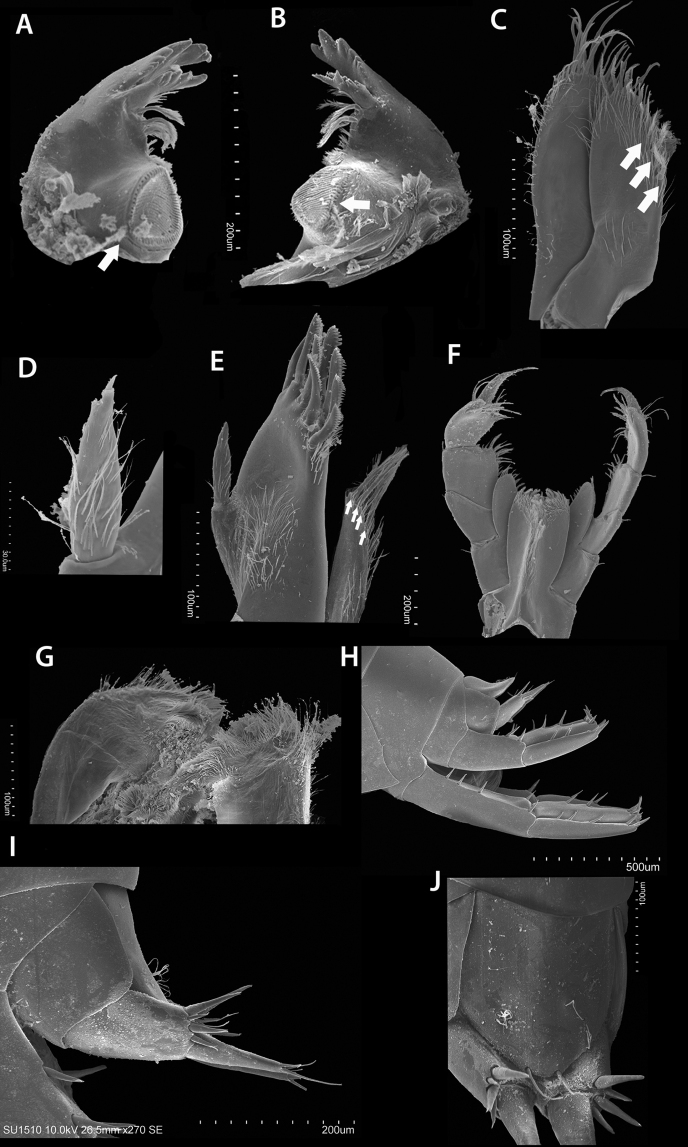
*Hyalella tepehuana* sp. nov., male buccal parts **A** left mandible **B** right mandible **C** maxilla 2 **D** palp maxilla 1 **E** maxilla 1 **F** maxilliped **G** lower lip **H** uropods 1–3 **I** uropod 3 **J** telson. Arrows show setae.

Figures 2 , 3 , 4 , 5 , 6 , 7 , 8

##### Etymology.

The specific epithet *tepehuana* refers to the great Tepehuan ethnic group, whose current settlement is in southern Durango. The name Tepehuan comes from Náhuatl and has two meanings: “owners of the hills”, *tepetl* (hill) and *huan* (possessive), and “winner of battles”, *tepehuani*.

##### Material examined.

Holotype male, body length 5.4 mm (CNCR 35295), from Tunal River in La Ferrería Durango, Mexico (23°57.905'N, 104°39.817'W) 12 June, 2016. Collectors: A. Marrón-Becerra and G. Rivas. Paratypes (*n* = 10 males, *n* = 10 females): males mean size 5.7 ± 0.6 mm, female body length 5.6 ± 0.6 mm (CNCR 35296, permanent slides and CNCR 35297 SEM preparations), same locality, date and collectors as holotype.

##### Type locality.

Mexico, Durango, Tunal River in La Ferrería: 23°57.905'N, 104°39.817'W (Fig. 1), above 1874 m a.s.l., scarce water but with high density of macroalgae.

##### Diagnosis.

Pleonite 1 and 2 with dorsoposterior carina. Coxa 4 excavated posteriorly. Eyes pigmented. Antenna 1 shorter than Antenna 2 without accessory flagellum. Antenna 2 less than one-half body length. Maxilla 1 palp short, reaching less than half distance between base of palp and tip of setae of outer plate, with one stout distal seta; inner plate slender with three or four strong pappose distal setae. Maxilla 2 inner plate with two or three strong pappose setae on inner margin. Gnathopod 1, propodus hammer-shaped, palm slope transverse, inner face with three or four pappose setae, comb scales on distoposterior and distoanterior border. Gnathopod 2, basis hind margin with two setae. Uropods without curved setae. Uropod 3, peduncle and ramus subequal in length, styliform. Telson slightly longer than wide, narrowing posteriorly, with two long simple setae widely separated.

##### Description of male.

***Body***, tergites of pleon 1 and 2 with dorsoposterior carina (Fig. 2A, B).

Mean body length of males: 5.7 ± 0.6 mm (*n* = 10).

Mean cephalothorax length: 0.6 ± 0.03 mm (*n* = 10).

Epimeral plates 1–3 acuminate.

***Coxae 1–3*** (Fig. 2A, B) subequal in shape, subrectangular, longer than wide; coxa 1 shorter than coxae 2 and 3; coxa 4 wider than coxae 1–3 with a deep posterior excavation; coxae 1–4 slightly overlapping anterior coxa, distal margin rounded with small setae widely separated, acumination absent. Coxae 5–7 reduced, shorter than coxae 5 and 6, bilobate except coxa 7; coxa 5 with two subequal lobes, posterior lobe slightly longer than anterior; coxa 6 with two unequal lobes, anterior lobe reduced; coxa 7 with a single lobe, anterior lobe absent.

***Head*** typically gammaridean (Fig. 2A, B), smooth surface, length less than combined length of the first two thoracic segments, reaching the half of the second pereionite, rostrum absent. Eyes pigmented, medium, rounded, located between insertions of antennae 1 and 2.

***Antenna 1*** (Figs 2A, B, 3A) less than one-half the body length, shorter than antenna 2 (80% length of A2), but longer than peduncle of antenna 2, reaching more than one-half of the third pereionite; peduncle longer than head, reaching beyond half of the length of first pereionite, article 1 and 2 subequal in length, article 1 wider than articles 2 and 3, article 2 longer and wider than article 3, article 3 shorter and thinner than articles 1 and 2, proportions (1.5:1.4:1), article 1 close to the middle length of the ventral surface with two short cuspidate setae, one smaller, and one cluster with three cuspidate setae at distal end; flagellum with 9–11 articles reduced gradually toward the distal portion, flagellum longer than peduncle; aesthetasc on flagellum, present on articles 4–11, 4–7 (one pair), 8–11 (one aesthetasc). Accessory flagellum absent.

***Antenna 2*** (Figs 2A, B, 3B) almost 1.2× longer than antenna 1, slightly longer than one-third body length, reaching half of the fifth pereionite; peduncle reaching the second pereionite, peduncle articles increase gradually in length and decrease in width, article 3 shorter but wider than articles 4 and 5, article 4 a little longer than the length of article 3, article 5 slightly longer than article 4; flagellum with 11–12 articles, slightly longer than peduncle, almost 1.25× the length; without aesthetasc on flagellum.

***Buccal parts***: upper lip (Fig. 3E) with distal margin rounded and numerous setules present, longer and more distant toward the lateral end, two setae plus accessory setae near the distal margin on both sides (left and right) symmetrically.

**Lower lip** (Figs 3F, 8G), outer lobes without notches or excavations; mandibular projection of outer lobes rounded; without inner lobes.

***Mandibles*** (Figs 3G, H, 8A, B) without palp, asymmetric. Incisor toothed, six to eight teeth present. Left lacinia mobilis similar to incisor process, with five or six teeth; setal row on left mandible with three or four main pappose setae plus accessory setae. Right mandible with six or seven teeth, lacinia mobilis reduced than the left one, with two pairs of asymmetrical, L-shaped teeth, fused at the base, proximal pair shorter than distal, inner margin denticulate; near the lacinia mobilis base, with one pair of setae; setal row with two main pappose setae plus accessory setae and with setulae near the molar process. Molar process large, cylindrical, and triturative; left mandible lateral view almost rectangular (Fig. 3G) and right mandible with a 60° angle (Fig. 3H); with accessory pappose setae present in both molars.

***Maxilla 1*** (Figs 3C, 8D, E) with short palp, vestigial, uniarticulate, longer than wide, distally pointed with one short and stout distal seta (Fig. 8D), palp length almost exceeds half of the distance between base of palp and base of seta on outer plate, but less than half the distance between base of palp and tip of seta on outer plate (Fig. 8E); inner plate slender, shorter than outer plate, with three or four pappose distal setae (two distal and one or two subdistal); outer plate with nine stout serrate setae (Fig. 8E).

***Maxilla 2*** (Figs 3D, 8C) with plates subequal in length, width, and shape; inner plate shorter and slender, with two or three pappose setae on mid-distal margin, and with seven shorter serrulate setae on distal margin; outer and inner plates with abundant setules.

***Maxilliped*** (Figs 3I, 8F) with inner plate longer than outer plate; distal margin slightly convex, almost flat, in both plates; inner plate distal margin with three cuspidate setae of equal size and with plumose setae; outer plate, inner and distal margins with numerous simple setae. Palp composed of four articles subequal in the maximum length; first article with three simple setae at the inner distal end and one pair at the outer distal end, one on each side; second article with numerous simple setae on the inner margin and three on outer distal end; third article with several setae on distal margin, distal end on the outer margin with three simple setae and comb setae; fourth article ungiform, longer than nail, with comb setae, inner margin near the distal half with three setae and one near nail base on outer margin; nail reaching almost two-thirds of the fourth article with serration at distal half.

***Gnathopod 1*** (Figs 4A, 6A–C) subchelated, hammer shaped, shorter than gnathopod 2. Basis elongated, maximum length close to 3× longer than the maximum width; near to the half of posterior margin with one seta, distal end with two clusters of two setae. Ischium short, almost as long as wide, length almost same as maximum width of basis and maximum length of merus; distal posterior end with two clusters of two setae. Merus longer than wide, almost half of the length of ventral surface with comb scales; distal margin with four setae. Carpus longer than wide, longer and slightly wider than propodus, with strong short and wide posterior lobe forming a scoop-like structure open to the inside; lobe, inner surface with three serrate setae, external surface on the distal half of lobe, near to the margin, with comb scales, anterior distal end with three setae. Propodus 1.5× longer than wide; inner surface near to the distal margin with four serrate setae (three in a row); distal anterior end with two clusters of approximately five setae; distal anterior and posterior surfaces with comb scales; palm transverse, posterior distal end with a robust seta and cup for dactyl. Dactyl claw-like; nail present; anterior surface near to the proximal half with one plumose seta, with comb scales over the anterior surface.

***Gnathopod 2*** (Figs 4B, 6D, E) subchelated; palm slightly oblique. Basis elongate, more than 3× longer than wide; posterior margin with two long setae. Ischium short, subquadrate, shorter than merus. Merus short; distal end of posterior margin with eight simple setae; distal half of the posterior inner and outer surfaces with comb scales. Carpus shorter than propodus; anterodistal end with two setae; posterior lobe scoop-like, elongate, length similar to the merus maximum length, almost 1.5× the width of merus, with several submarginal pappose setae and comb scales. Propodus robust, almost 1.5× as long as wide, subrectangular; palm slightly shorter than posterior margin, slope slightly irregular, with some long simple setae, several short, and several medium setae; distal margin of palm with one truncated process and presence of one slightly posterior excavation at base, near to the insertion of dactyl; palm posterior distal end with two strong setae, comb scales and cup for dactyl. Dactyl claw-like, congruent with palm, without comb scales; outer margin proximal third with a plumose seta; inner margin crenulate.

***Pereopods 3–7*** (Figs 4E–I, 7D–I) simple, gradually longer posteriorly. Pereopod 5 shorter than pereopods 4 and 6.

***Pereopod 3*** (Figs 4E, 7D) with basis elongate; mid-posterior margin with two simple setae; anterodistal and posterodistal ends with simple setae. Ischium subquadrate; posterodistal end with one pair of setae. Merus longer than ischium (more than twice the length); posterior margin with three setae; anterior margin with one seta; anterodistal and posterodistal ends with one cluster of four setae. Carpus shorter and slenderer than merus; posterior margin with four stout setae; posterodistal end with at least five slender setae, longer than the ones from posterior margin; anterodistal end with at least two setae. Propodus almost as large as the posterior margin of merus, slenderer than carpus; posterior margin with eight setae; anterodistal end with three simple setae. Dactyl claw-like; nail present; first proximal third of the anterior margin with one plumose seta; posterior margin with one simple seta close to the nail.

***Pereopod 4*** (Figs 4F, 7E) similar in shape to pereopod 3 but slightly longer; coxa 4 wider than coxa 3, with a posterior excavation; basis posterior margin with one simple seta.

***Pereopods 5–7*** (Figs 4G–I, 7F–H) similar in shape; basis posterior lobe rounded and denticulate. Pereopod 7 (Fig. 7I) with basis lobe widely expanded, almost reaching ischium distal margin; wider than lobes of pereopods 5 (Figs 4G, 7F) and 6 (Figs 4H, 7G); width almost 0.75× width of basis (measured at cleft between basis and basis lobe); posterior margin with 14 serrations, each with one setule but one or two serrations with one stout seta in the distal margin; anterior margin with three stout setae and one at distal end.

***Pleopods 1–3*** (Fig. 5E) not modified, biramous, elongated, rami multi-annulated, with numerous plumose setae; inner margin of peduncle with two short retinacula (coupling hooks) at distal end.

***Uropod 1*** (Figs 5A, 8H) longer than uropod 2 (Fig. 8H); peduncle longer than rami, proximal half of the dorsal margin with three or four dorsal setae, inner and outer distal end with one seta; rami subequal, inner ramus slightly shorter, with two dorsal setae and four distal setae, outer ramus with three dorsal and three distal setae; male without curved setae on inner ramus.

***Uropod 2*** (Figs 5B, 8H) longer than peduncle of uropod 1; peduncle as long as rami, with two dorsal setae over the distal half and one at distal end; rami subequal, inner ramus with two dorsal and four distal setae, outer ramus with two or three dorsal and three distal setae.

***Uropod 3*** (Figs 5C, 8H–I) slightly shorter than peduncle of uropod 2; peduncle rectangular, wider than ramus with four strong distal setae of variable length, inner ramus absent, outer ramus uniarticulate slender, slightly shorter than peduncle, basal width near 3× the apex of ramus, with three or four slender apical setae and one connate seta.

***Telson*** (Figs 5D, 8J) entire, slightly longer than wide, narrowing posteriorly, with two long simple setae widely separated; outer surface bearing two clusters of three plumose setae near the half distal portion, close to the margin, symmetrically distributed.

***Coxal*** gills sac-like, present on segments 2–6 (Fig. 6D). Sternal gills tubular, present on segments 3–7.

**Female** (Fig. 2C). Similar to male. Gnathopod 1 (Figs 4C, D, 7A–C) with carpus with five setae on the inner face lobe; propodus with four setae in a row over the inner face. Gnathopod 2 smaller than male gnathopod 2, parachelated, palm reverse oblique; basis posterior margin with two setae; propodus slightly longer than twice its maximum width, outer face with three setae in a row and three large setae near the palm, anterior and posterior distal half with comb scales. Pereonite 2 with one anterior excavation or notch for the amplexus. Pereopod 7 lobe with 13 serrations and setules, and two stout setae on the distal margin. Oostegites subtriangular, with setae curled on the margins, reaching almost one-half length of merus (Fig. 4C).

Intraspecific variation: Maxilla 1, inner plate usually with four setae, the smaller adults could have three setae and the young ones two setae. Maxilla 1 palp, length quite variable during the molt process. Maxilla 2 inner plate with two or three setae, even in the same organism.

##### Habitat.

Freshwater, epigean.

##### Distribution.

La Ferrería, Durango, Tunal river (23°57.905'N, 104°39.817'W).

##### Remarks.

*Hyalella tepehuana* sp. nov. is the first species described from northern Mexico. It is easily distinguished from other species of *Hyalella* from the USA, Mexico, and the Caribbean by the atypical number of setae on the inner plate of maxilla 1 (four setae) and maxilla 2 (three setae), and by the shape of the telson. The species morphologically most similar to *H. tepehuana* sp. nov. is *H. faxoni* Stebbing, 1903 from the Volcan Barva in Costa Rica, but *Hyalella tepehuana* sp. nov. differs by the presence of dorsal processes on pereionites 1 and 2, and by the following characters: number of articles on antennae 1 and 2 (9–11 and 11 or 12, respectively, in *H. tepehuana* sp. nov. versus 12 and 15–17, respectively, in *H. faxoni*); number of setae on propodus inner face of gnathopod 1 (four in *H. tepehuana* sp. nov. versus five in *H. faxoni*); number of setae on the posterior basis of male gnathopod 2 (two in *H. tepehuana* sp. nov. versus four in *H. faxoni*); shape of uropod 3 (styliform in *H. tepehuana* sp. nov. versus globose in *H. faxoni*); and telson shape (longer than wide in *H. tepehuana* sp. nov. versus quadrate and wider than long in *H. faxoni*). Furthermore, in the new species, the fourth article (= dactyl) of maxilliped is more slender than in *H. faxoni*, according to González and Watling (2002b) (Table 1). Unlike *H. faxoni*, *Hyalella tepehuana* sp. nov. and *H. azteca* have a dorsal process on perionites 1 and 2. The differences between these last two species are considerable, based mainly on the morphology of palp of maxilla 1, the number of setae on inner plate of maxilla 1 and maxilla 2, the number of setae on uropods, the shape of the telson and the distance between the distal setae on the telson, according to the redescription by González and Watling (2002a) (Table 1). These differences seem sufficient to distinguish *Hyalella tepehuana* sp. nov. from *H. azteca* and other species from North America.

**Table 1.** Morphological differences of males and females among *Hyalella azteca* (De Saussure, 1858) (based in redescription by González and Watling 2002a and the type material), *Hyalella faxoni* Stebbing, 1903 (based in the redescription by González and Watling 2002b), and *Hyalella tepehuana* sp. nov.

**Character**	***Hyalella azteca* (De Saussure, 1858)**	***Hyalella faxoni* Stebbing, 1903**	***H. tepehuana* sp. nov.**
Size (mm)	7.8	8.7	5–6.75
Dorsal process in pereionites 1–2 (mucronations)	yes	no	yes
Maxilla 1, number of pappose setae on the inner plate	3	4	3–4
Maxilla 1, palp apical stout setae	no	yes	yes
Maxilla 2, inner plate pappose setae	2	3	2–3
Mandibles, lacinia mobilis number of teeth	5	5	5–6
Antenna 1, number of flagellum articles	7	12	9–11
Antenna 1, number of flagellum articles	8	15–17	11–12
Uropod 3	styliform	globose	styliform
Male gnathopod 2, hind margin setae	2	4–6	2
Male gnathopod 1, carpus lobe, inner face, number of pappose setae	1–3	1–3	3
Male gnathopod 1, propodus, inner face, number of pappose setae	4	5	3–4
Female gnathopod 1, propodus palm	reverse oblique	slightly reverse oblique	slightly reverse oblique
Uropod 1, outer ramus dorsal setae	2	3	3
Telson	width ≈ length, apically pointed with two apposed long simple setae	width > length, quadrate with two short widely apart setae	width < length, apically narrowed (semitriangular) with two long widely apart simple seta

Due to the subtle variations within species, and lack of morphological studies and formal descriptions, the identification of species of *Hyalella* in North America is complex. Hence, the new characters proposed by Soucek et al. (2015) to distinguish species are useful: proportion of length of ramus uropod 3 versus the length of stout setae in peduncle, and proportion of gnathopod 2 merus width versus carpus lobe width. The ramus of uropod 3 in *Hyalella tepehuana* sp. nov. is larger than peduncle stout setae. The relative proportions of the merus width and carpus lobe of the ganthopod 2 of *H. tepehuana* sp. nov. are similar as *H. spinicauda* in Michigan and Wisconsin, USA, and some localities in Canada, and different from the proportions found in *H. azteca* (1.5×).

*Hyalella tepehuana* sp. nov. is also similar to the recently described *H. wakulla* Drumm & Knight-Gray, 2019, from Florida, USA. These two species have a similar body length, about 5.5 mm, by which they may be considered to be smaller ecomorphs; however, the main differences between these two species are the number of articles in antennae 1 and 2, and the number of setae on the buccal parts: *Hyalella tepehuana* sp. nov. has more articles in antenna 1 (9–11) and antenna 2 (11–12) while *H. wakallua* has fewer articles in antenna 1 (eight) and antenna 2 (nine). The new species also bears a maximum of four setae on the inner plate of maxilla 1 and bears setules on the palp, whereas, *H. wakulla* bears three setae on inner plate of maxilla 1, and the palp lacks setules. Also, *H. tepehuana* sp. nov. has a maximum of three pappose setae on the inner plate of maxilla 2, while *H. wakulla* has only two, and the maxilliped fourth article in *H. tepehuana* sp. nov. has fewer than four subterminal setae on the medial margin in contrast to *H. wakulla*, which has a maximum of four subterminal setae in adults. We consider the differences presented here sufficient to consider *Hyalella tepehuana* sp. nov. as a new species.

### Key to the species of Hyalella (Hyalella) in North America, Central America and the Caribbean region^1^

**Table:** 

1	Eyes absent	**2**
–	Pigmented eyes present	**3**
2	Antenna 1 is longer than antenna 2; sternal gills on pereonites 3–7; telson with four distal setae	***H. muerta***
–	Antenna 1 shorter than antenna 2; sternal gills on pereonites 2–7; telson without distal setae	***H. cenotensis***
3	Body with dorsal mucronations	**9**
–	Body without dorsal mucronations	**4**
4	Ramus of uropod 3 is vestigial or robust, subequal or shorter than peduncle	**5**
–	Ramus of uropod 3 slender, subequal or longer than peduncle	**6**
5	Ramus of male uropod 3 robust, with seven apical spines	***H. sandra***
–	Ramus of male uropod 3 vestigial, with two to four spines	***H. meraspinosa***
6	Antenna 1 and 2 are subequal in length (antenna 1 slightly shorter)	**7**
–	Antenna 2 is nearly twice the length of antenna 1	***H. longicornis***
7	Hind margin of merus of pereopods 3 and 4 with long setae; telson with two closely apical setae	***H. caribbeana* (*H. squamosa*, material needs revision and redescription, but the main differences seem to be the length of antennae and the chaetotaxia in the gnathopods (basis, carpus, and propodus))**
–	Hind margin of article 4 of pereopods 3 and 4 with short setae; telson with two long, broadly-spaced, apical setae	**8**
8	Maxilla 1 inner plate with two setae, pereiopod 7 basis lobe ventral margin with three stout setae; pereiopod 7 basis anterior margin half distal margin with short stout setae (4); uropod 2 ramus with two dorsal setae; maxilla 2 with serrate setules	***H. cheyennis* (*H. inermis*, material needs revision and redescription, but the main difference seems to be the maxilla 2 with serrate setules according to Bueno et al. (2019))**
–	Maxilla 1 inner plate with more than two setae (4), pereiopod 7 basis lobe ventral margin without stout setae; pereiopod 7 basis anterior margin half of proximal and distal margin with short stout setae (7); uropod 2 peduncle ramus with three dorsal setae; maxilla 2 probably without serrate setules	***H. faxoni***
9	Inner plate of maxilla 1 narrow, with two to five apical plumose setae	**10**
–	Inner plate of maxilla 1 is broad, subtriangular, with two or three apical plumose setae, followed closely by 22–30 similar medial setae	***H. montezuma***
10	Antenna 1 is longer than half the length of antenna 2, and only first or first two abdominal segments bearing dorsal mucronations	**11**
–	Antenna 1 is less than half the length of antenna 2, with all three abdominal segments bearing dorsal mucronations	***H. texana***
11	Gnathopod 2 of males, carpus posterior lobe is about as long as width of merus; in pereopod 7, the distal/ventral margin of basis posterior lobe, dentate or not, with one or two very small setae if any	**13**
–	Gnathopod 2 of males, carpus posterior lobe approximately 1.5× as long as width of merus, pereiopod 7, distal/ventral margin of basis posterior lobe dentate with at least three stout setae	**12**
12	Pereiopod 3 posterior margin with one setae; pereiopod 5 merus and carpus length subequal; pereopod 7 with distal/ventral margin of the basis posterior lobe strongly dentate, with two or more (five) relatively long spines; telson distal margin acute with two apposed setae; female gnathopod 1; carpus inner face with two setae; mandible incisor with six teeth	***H. azteca***
–	Pereiopod 3 posterior margin with two setae; pereiopod 5 merus longer than carpus; pereopod 7 with distal/ventral margin of the basis posterior lobe strongly dentate, and with one or two relatively long spines (fewer than three), distal end of telson narrowing , distal margin rounded or truncated with two widely apart setae; female gnathopod 1, carpus inner face with four setae; mandible incisor with seven teeth	***H. tepehuana* sp. nov.**
13	Gnathopod 2 propodus in males: palm with a distinct angle step (visible under high power), tip of dactyl approximately aligns vertically with distal end of posterior lobe of carpus; telson distal setae is separated, short, and at least as stout as setae on uropod 3 ramus	***H. spinicauda***
–	Gnathopod 2 propodus in males: palm without a distinct angle step or notch, tip of dactyl aligning vertically well beyond (posteriorly) distal end of posterior lobe of carpus; telson terminal setae clearly thinner and longer than setae on uropod 3 ramus	**14**
14	Telson with two long and slender apposed setae; uropod 3 ramus approximately as long as or slightly longer than the longest seta on peduncle; pereopod 7 posterior lobe ventral margin without stout setae; maxilla 1, inner plate with two pappose setae; maxilliped nail short, less than half the length of palp article 4	***H. wellborni***
–	Telson with two long and slender setae widely separated; uropod 3 ramus longer than the longest setae on peduncle; pereopod 7 posterior lobe ventral margin with one stout setae; maxilla 1, inner plate with three pappose setae; maxilliped nail long, more than half length of palp article 4	***H. maya***

## Supplementary Material

XML Treatment for Hyalella tepehuana

## Footnotes

modified from Baldinger 2004; Marrón-Becerra et al. 2014; Soucek et al. 2015; Marrón-Becerra et al. 2018.
